# Serum IgG4 levels at diagnosis can predict unfavorable outcomes of untreated patients with IgG4-related disease

**DOI:** 10.1038/s41598-021-92814-8

**Published:** 2021-06-25

**Authors:** Ichiro Mizushima, Masahiro Konishi, Hajime Sanada, Kazuyuki Suzuki, Akari Takeji, Takeshi Zoshima, Satoshi Hara, Kiyoaki Ito, Hiroshi Fujii, Kazunori Yamada, Mitsuhiro Kawano

**Affiliations:** Department of Rheumatology, Kanazawa University Hospital, 13-1, Takara-machi, Kanazawa, Ishikawa 920-8640 Japan

**Keywords:** Immunology, Biomarkers

## Abstract

The outcomes of patients with immunoglobulin G4 (IgG4)-related disease (IgG4-RD) who are not treated are unclear. This study aimed to clarify these outcomes and identify the factors related to them. We retrospectively evaluated various clinical features including laboratory data and involved organs at diagnosis in 107 patients with IgG4-RD, who were followed up for more than 6 months, at a single center in Japan. We compared the clinical features of the 27 untreated patients with those of the 80 patients treated with glucocorticoid. The patient outcomes were investigated, and logistic regression analysis was performed to identify factors related to them. The patients comprised 73 men and 34 women (median age 67 years). The untreated patients had significantly lower IgG4-RD responder index (9 vs. 12) and fewer affected organs (1 vs. 3) than did those treated with glucocorticoid. Of these 27 patients, 8 experienced deterioration of IgG4-RD after the diagnosis. In the age- and sex-adjusted logistic regression analysis, serum IgG4 elevation (per 100 mg/dL, odds ratio 1.194, 95% confidence interval 1.017–1.402) was the only significant factor related to disease deterioration in untreated patients with IgG4-RD, whereas not serum IgG4 levels (per 100 mg/dL, odds ratio 0.995, 95% confidence interval 0.921–1.075) but history of allergy (OR 3.134, 95% confidence interval 1.094–8.977, *P* = 0.033) related to deterioration in patients who underwent treatment. Serum IgG4 levels may be a useful predictor of unfavorable outcomes in untreated patients with IgG4-RD, who tend to have fewer affected organs and lower IgG4-RD responder index.

## Introduction

Immunoglobulin G4 (IgG4)-related disease (IgG4-RD) is a systemic fibro-inflammatory disease that can affect almost all organs of the body^1,2^. In IgG4-RD, spontaneous, or at least temporary, remissions without treatment have been reported, and watchful waiting may be appropriate in certain patients with asymptomatic and inactive disease^3^.

Indeed, in type 1 autoimmune pancreatitis (AIP), it was reported that spontaneous remissions (SR) without treatment were observed in 55.7–65.0% of patients^4,5^. The suggested predictors for SR included absence of serum IgG4 elevation, female gender, and stent placement for jaundice. On the other hand, unfavorable events including symptomatic, radiological, or functional exacerbation of the organ involved or relapse occurred in 50–70% of AIP patients without treatment^5,6^. New onset of diabetes mellitus and extensive multi-organ involvement were reported as predictors for unfavorable events in untreated patients^5^.

However, the outcomes of patients with IgG4-RD, especially those except for AIP, who do not undergo treatment are still unclear. This state of affairs prompted us to undertake the present study to clarify the outcomes of untreated patients with IgG4-RD and the factors related to them.

## Results

### Baseline patient profiles

The profiles of the 107 IgG4-RD patients are listed in Table 1. The median follow-up period after the start of therapy or observation without therapy was 63 months [interquartile range (IQR) 25, 85]. At diagnosis, their median serum IgG4 level was 486 mg/dL (IQR 220, 991). Involvement of the salivary gland was observed in 58% of patients, eye in 47%, pancreas in 26%, perivasculature in 27%, kidney in 21%, lung in 25%, and retroperitoneum in 8%. Twenty-seven patients were followed up without treatment after the initial diagnosis according to the decision of each attending physician. Of the 27 patients, 19 had a history of allergy including allergic rhinitis in 7, bronchial asthma in 7, urticaria in 4, atopic dermatitis in 2, drug allergy in 2, and milk allergy in 1. However, none of the patients including the 19 in this study received any allergen-specific immunotherapy before or at the time of diagnosis or during the observation periods. Compared with the 80 patients with treatment, these 27 patients had a significantly lower IgG4-RD responder index [9 (6, 15) vs. 12 (9, 18), *P* = 0.048], fewer affected organs [1 (1, 3) vs. 3 (2, 4), *P* = 0.001], and lower frequency of ophthalmic and renal parenchymal lesions (26% vs. 54%, *P* = 0.015, and 4% vs. 26%, *P* = 0.012, respectively).

**Table 1:** Baseline clinical characteristics of 107 patients with IgG4-related disease.

	Overall	Treatment		
	n = 107	(−)	(+)	*P* value*
		n = 27	n = 80	
Age	67 (59, 73)	70 (60, 78)	66 (59, 72)	0.144
Male gender (%)	68	78	65	0.243
Allergy (%)	56	70	51	0.116
Serum IgG4 level (mg/dL)	486 (220, 991)	361 (187, 1,040)	495 (254, 975)	0.507
Serum IgG level (mg/dL)	1951 (1531, 2872)	1960 (1512, 2655)	1950 (1535, 2936)	0.637
Serum IgE level (IU/mL)	486 (190, 1231)	755 (122, 1764)	474 (235, 1036)	0.670
Serum IgG4/IgE ratio	0.99 (0.38, 3.24)	0.54 (0.22, 4.28)	1.11 (0.40, 3.15)	0.494
Serum C3 level (mg/dL)	90 (76, 110)	90 (76, 106)	94 (76, 110)	0.810
Serum C4 level (mg/dL)	22 (14, 27)	22 (19, 28)	21 (14, 27)	0.363
Serum CH50 level (IU/L)	51 (38, 58)	50 (39, 56)	51 (35, 59)	0.976
Serum CRP level (mg/dL)	0.1 (0.0, 0.3)	0.1 (0.0, 0.2)	0.1 (0.0, 0.3)	0.599
Serum creatinine level (mg/dL)	0.79 (0.64, 1.00)	0.75 (0.65, 0.94)	0.80 (0.60, 1.05)	0.415
Eosinophil count (/μL)	204 (118, 426)	180 (119, 598)	230 (118, 422)	0.766
RF positivity (%)	20	17	21	0.772
ANA positivity (%)	14	11	15	0.755
Pancreatic lesion (%)	26	19	29	0.448
Salivary gland lesion (%)	58	67	55	0.369
Ophthalmic lesion (%)	47	26	54	0.015
Renal lesion (%)	21	4	26	0.012
Vascular lesion (%)	27	30	26	0.804
Retroperitoneal lesion (%)	8	4	10	0.444
Lung lesion (%)	25	11	30	0.072
Number of involved organs	2 (1, 4)	1 (1, 3)	3 (2, 4)	0.001
IgG4-RD responder index	12 (9, 18)	9 (6, 15)	12 (9, 18)	0.048

Conversion factor for Cr: mg/dL to μmol/L, × 88.4. Data are presented as median [interquartile range (IQR1, IQR3)]. *Treatment (+) versus Treatment (−).

*ANA* anti-nuclear antibody, *CRP* C-reactive protein, *IgG* immunoglobulin G, *IgG4* immunoglobulin G4, *IgG4-RD* immunoglobulin G4-related disease, *IgE* immunoglobulin E, *PSL* prednisolone, *RF* rheumatoid factor.

### Spontaneous improvement (SI) of IgG4-RD

During the clinical course, 6 of the 27 untreated patients experienced SI (Table 2). Renal pelvic lesion improved spontaneously in 2 patients, and lacrimal gland lesion, submandibular gland lesion, pancreas, retroperitoneum, and periaortic lesion in one each. In the age- and sex-adjusted logistic regression analysis, male gender [vs. female, odds ratio (OR) 0.064, 95% confidence interval (CI) 0.006–0.644, *P* = 0.020] and serum C3 levels (OR 1.090, 95% CI 1.005–1.182, *P* = 0.039) were significant factors negatively and positively related to SI, respectively, in untreated patients with IgG4-RD (Table 3).

**Table 2:** Baseline clinical data of 27 untreated patients with IgG4-related disease.

No	IgG4 (mg/dL)	IgG (mg/dL)	IgE (IU/mL)	Hypocomple-mentemia	Eosinophil count (/µL)	Allergy	Involved organs	Number of involved organs^§^	IgG4-RD RI	SI	Deterio-ration
1	164	1960	1621	−	197	+	Sa, La	2	9	−	−
2	2150	3310	74	−	177	+	Sa, La, *K (#M)	3	12	+	+
3	354	1963	764	−	138	−	A	1	9	−	−
4	196	1487	447	−	191	+	A, *RF	2	15	+	−
5	187	1317	134	−	21	+	Sa, *La	2	9	+	−
6	73.1	1277	49	−	180	+	Sa	1	3	−	−
7	1230	2655	1668	−	1014	+	Sa, K, L, S	4	18	−	−
8	565	1732	135	−	113	+	P	1	12	−	−
9	2150	3751	4423	−	149	+	Sa	1	9	−	−
10	1520	2938	48	−	79	−	#Sa (#La, #K, #A)	1	6	−	+
11	361	1936	8	+	119	−	Sa, Ly (#La, #P, #K, #A)	2	9	−	+
12	1040	2127	227	−	249	−	#Sa, A, Ph (#La, #RF)	3	15	−	+
13	632	1934	328	−	70	+	La	1	6	−	−
14	997	2078.7	1730	−	150	−	A, Bi (#P)	2	15	−	+
15	254	1559.6	745	−	290	−	Sa, La, L, Ly	4	15	−	−
16	374	1413.9	883	−	180	+	K	1	6	−	−
17	1160	3506	2328	−	68	+	L (#Sa, #P, #A)	1	18	−	+
18	1740	4298	324	−	959	+	Sa (#A, #Pr)	1	15	−	+
19	292	2141	87	−	362	−	P	1	6	−	−
20	313	2237	1867	−	70	−	*Sa, La, A, Ly, Pec	5	24	+	−
21	534	1674	86	−	598	+	Sa, La, *P, *K	4	15	+	−
22	136	1419	1247	−	188	+	Sa	1	6	−	−
23	133	983	1364	−	829	+	Sa	1	3	−	−
24	185	1924	32,313	−	2952	+	Sa	1	6	−	−
25	297	2213	2042	−	772	+	Sa, P, K	3	12	−	−
26	99.8	1512	NA	−	121	+	Bi (#*A)	1	6	+	+
27	969	3635	4042	−	2262	+	K, Bi	2	12	−	−

*A* aortic/arterial lesion, *Bi* bile duct lesion, *IgG* serum immunoglobulin G levels, *IgG4* serum immunoglobulin G4 levels, *IgG4-RD RI* immunoglobulin G4-related disease responder index, *IgE* serum immunoglobulin E levels, *K* kidney lesion, *L* lung lesion, *La* lacrimal grand lesion, *Ly* lymph node lesion, *M* mammary lesion, *P* pancreas lesion, *Pec* pericarditis, *Ph* pharyngeal mass, *Pr* prostate lesion, *RF* retroperitoneal fibrosis, *S* skin lesion, *Sa* salivary gland lesion, *SI* spontaneous improvement.

*Organ in which spontaneous improvement occur. #Organ in which deterioration occur. ( )De novo organ involvement.

^§^Number of involved organs at the first diagnosis of IgG4-RD.

**Table 3:** Odds ratio for risk of spontaneous improvement of IgG4-RD: unadjusted and age- and sex-adjusted logistic regressions.

Variable	Untreated patients					
	Unadjusted			Age- and sex-adjusted		
	OR	95% CI	*P* value	OR	95% CI	*P* value
Age (years)	0.943	0.869–1.024	0.161	0.973	0.886–1.069	0.574
Male gender	0.053	0.006–0.493	0.010	0.064	0.006–0.644	0.020
Allergy	2.500	0.243–25.717	0.441	1.490	0.092–24.090	0.779
Serum IgG4 level (100 mg/dL)	0.967	0.825–1.135	0.684	0.967	0.806–1.160	0.718
Serum IgG level (100 mg/dL)	0.946	0.831–1.077	0.399	0.966	0.826–1.131	0.667
Serum IgE level (100 IU/mL)	0.925	0.804–1.064	0.276	0.991	0.921–1.066	0.807
Serum IgG4/IgE ratio	1.018	0.943–1.100	0.646	1.015	0.901–1.145	0.804
Serum C3 level (mg/dL)	1.053	1.004–1.106	0.035	1.090	1.005–1.182	0.039
Serum C4 level (mg/dL)	1.024	0.936–1.119	0.607	1.105	0.960–1.272	0.163
Serum CH50 level (IU/L)	1.055	0.965–1.153	0.243	1.117	0.968–1.290	0.130
Serum CRP level (mg/dL)	1.299	0.069–24.497	0.861	6.467	0.245–170.80	0.264
Serum Cr level (mg/dL)	0.021	0.000–6.486	0.186	7.379	0.024–2247.9	0.493
Eosinophil counts (100/μL)	0.813	0.532–1.242	0.338	0.821	0.462–1.457	0.499
Number of involved organs	2.173	0.988–4.775	0.053	1.925	0.746–4.918	0.176
IgG4-RD responder index	1.147	0.949–1.388	0.156	1.222	0.942–1.584	0.130

Conversion factor for Cr: mg/dL to μmol/L, × 88.4

*ANA* anti-nuclear antibody, *Cr* creatinine, *CRP* C-reactive protein, *IgG* immunoglobulin G, *IgG4* immunoglobulin G4, *IgE* immunoglobulin E.

### Deterioration of IgG4-RD

Of the 27 untreated patients, 8 experienced deterioration of IgG4-RD 37.5 (IQR 14.5, 81.5) months after the diagnosis (Table 2). De novo organ involvement was observed in all 8 patients, 2 of whom concurrently suffered exacerbation of the organs involved. Two patients (patient number 2 and 26 in Table 2) experienced both SI and deterioration during the observation periods. One patient (number 2) experienced SI of the renal pelvic lesion earlier, and de novo organ involvement of a mammary gland later. The other patient (number 26) experienced de novo organ involvement of the periaorta earlier, and then had SI of the same periaortic lesion later. Thus, because SI and deterioration occurred metachronously in both patients, we considered that they had experienced SI and deterioration as independent events during the respective observation periods. Of the 80 treated patients, 25 experienced deterioration of IgG4-RD 31 (IQR 13, 63) months after the diagnosis. De novo organ involvement was observed in 9 patients, 2 of whom concurrently suffered exacerbation of the organs involved. Exacerbation of the organs involved without de novo organ involvement was observed in the remaining 16 patients.

In the age- and sex-adjusted logistic regression analysis, serum IgG4 elevation (per 100 mg/dL, OR 1.194, 95% CI 1.017–1.402, *P* = 0.030) was the only significant factor related to disease deterioration in untreated patients with IgG4-RD, whereas serum IgG4 levels did not relate to deterioration in patients who underwent treatment (per 100 mg/dL, OR 0.995, 95% CI 0.921–1.075, *P* = 0.901). On the other hand, history of allergy (OR 3.134, 95% CI 1.094–8.977, *P* = 0.033) was the only significant factor related to deterioration of disease in treated patients with IgG4-RD (Table 4).

**Table 4:** Odds ratio for risk of deterioration of IgG4-RD: unadjusted and age- and sex-adjusted logistic regressions.

Variable	Untreated patients						Treated patients					
	Unadjusted			Age- and sex-adjusted			Unadjusted			Age- and sex-adjusted		
	OR	95% CI	*P* value	OR	95% CI	*P* value	OR	95% CI	*P* value	OR	95% CI	*P* value
Age (years)	1.051	0.966–1.143	0.251	1.047	0.958–1.143	0.314	0.960	0.916–1.007	0.098	0.964	0.918–1.012	0.140
Male gender	2.500	0.243–25.717	0.441	1.871	0.166–21.054	0.612	0.569	0.215–1.510	0.258	0.655	0.240–1.789	0.409
Allergy	0.342	0.076–1.532	0.161	0.310	0.051–1.877	0.202	3.578	1.284–9.965	0.015	3.134	1.094–8.977	0.033
Serum IgG4 level (100 mg/dL)	1.197	1.021–1.404	0.027	1.194	1.017–1.402	0.030	0.989	0.916–1.067	0.766	0.995	0.921–1.075	0.901
Serum IgG level (100 mg/dL)	1.113	0.999–1.239	0.052	1.104	0.987–1.236	0.084	1.007	0.966–1.050	0.734	1.020	0.976–1.066	0.378
Serum IgE level (100 IU/mL)	0.951	0.861–1.051	0.323	0.929	0.828–1.041	0.206	0.997	0.966–1.028	0.828	1.005	0.973–1.039	0.751
Serum IgG4/IgE ratio	1.470	0.874–2.474	0.147	1.690	0.908–3.147	0.098	1.006	0.989–1.023	0.520	1.004	0.986–1.022	0.654
Serum C3 level (mg/dL)	0.973	0.931–1.018	0.237	0.978	0.934–1.025	0.354	1.001	0.986–1.017	0.850	0.996	0.980–1.013	0.658
Serum C4 level (mg/dL)	0.955	0.873–1.044	0.312	0.950	0.865–1.044	0.288	0.989	0.947–1.032	0.609	0.976	0.931–1.023	0.319
Serum CH50 level (IU/L)	0.962	0.900–1.028	0.255	0.959	0.893–1.031	0.255	1.000	0.976–1.025	0.989	0.995	0.968–1.022	0.696
Serum CRP level (mg/dL)	0.157	0.002–11.902	0.402	0.005	0.000–7.321	0.154	0.810	0.475–1.381	0.439	0.903	0.530–1.540	0.708
Serum Cr level (mg/dL)	0.864	0.027–27.692	0.934	0.091	0.001–12.694	0.342	1.150	0.670–1.976	0.612	1.472	0.773–2.806	0.240
Eosinophil counts (100/μL)	0.878	0.675–1.141	0.330	0.854	0.630–1.157	0.307	1.139	0.971–1.335	0.110	1.164	0.982–1.380	0.079
Number of involved organs	0.827	0.393–1.744	0.619	0.825	0.372–1.830	0.635	0.889	0.654–1.209	0.454	0.930	0.665–1.301	0.672
IgG4-RD responder index	1.070	0.908–1.260	0.421	1.050	0.870–1.268	0.611	0.952	0.883–1.027	0.203	0.961	0.885–1.044	0.348

Conversion factor for Cr: mg/dL to μmol/L, × 88.4

*ANA* anti-nuclear antibody, *Cr* creatinine, *CRP* C-reactive protein, *IgG* immunoglobulin G, *IgG4* immunoglobulin G4, *IgE* immunoglobulin E.

**Figure 1:**
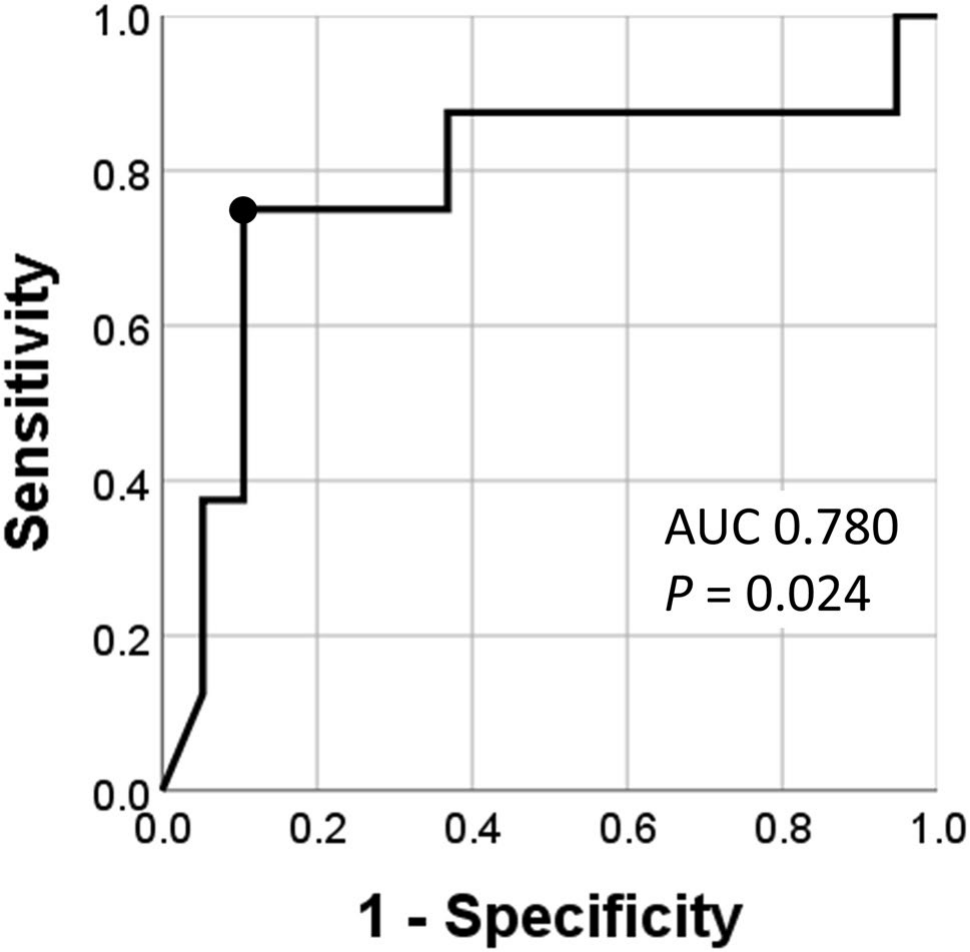
Receiver operating characteristic curve (ROC) analysis. ROC curve to identify the appropriate serum IgG4 level cutoffs for the prediction of disease deterioration shows that a serum IgG4 level > 983 mg/dL yields sensitivity of 75.0% and specificity of 89.5% (circle). *AUC* area under the curve.

To help determine the ability of the serum IgG4 level to predict reliably disease deterioration in untreated patients, and to identify its appropriate cut-off, we used an receiver operating characteristic (ROC) curve. The area under the ROC curve was 0.780 ± 0.115 (95% CI: 0.554–1.000, *P* = 0.024). We found that a serum IgG4 level of more than 983 mg/dL was the most appropriate cut-off that yielded a sensitivity of 75.0% and a specificity of 89.5% in predicting disease deterioration in untreated patients (Fig. 1).

## Discussion

The present study, which included patients with various organ involvement mainly salivary gland and ophthalmic lesions, showed that high serum IgG4 levels could be a useful predictor of unfavorable outcomes in untreated patients with IgG4-RD, who tend to have fewer affected organs and lower IgG4-RD responder index than treated patients. These results were different from those in the previous studies showing that new onset of diabetes mellitus and extensive multi-organ involvement were predictors of unfavorable events in untreated patients with AIP^5^. On the other hand, not serum IgG4 levels but the presence of allergic disease was related to deterioration in patients who underwent treatment in this study, consistent with the results of the previous study^7^. Because the natural course without treatment has rarely been evaluated in IgG4-RD except for AIP, these results might provide novel hints to the management of IgG4-RD without treatment and lead to significant advances.

In interpreting the results of the present retrospective study, we need to consider the characteristics of the analyzed untreated patients. Compared with treated patients, untreated ones had significantly lower IgG4-RD responder index and fewer affected organs despite any significant difference in serum IgG4 levels. In the international consensus guidance statement on the management and treatment of IgG4-RD, watchful waiting was described as possibly appropriate in certain patients with asymptomatic and inactive disease^3^. Involvement of multiple organs can be regarded as one indicator of disease activity. Therefore, it seems that decisions regarding treatment initiation were made according to the guidance in the present study. Such decisions, however, resulted in insufficient analysis regarding SI and deterioration of IgG4-RD during the natural clinical course in patients with higher disease activity.

Although spontaneous, or at least temporary, improvement without treatment has been reported in IgG4-RD, its prevalence and predictors remain to be clarified except for AIP. In AIP, it was reported that 55.7–65.0% of patients experienced SR and absence of serum IgG4 elevation, female gender, and stent placement for jaundice were significantly related to SR^4,5^. In the present study in which especially many patients with salivary and ophthalmic lesions were investigated, SI occurred in 6 (22.2%) of the 27 patients and was significantly related not to serum IgG4 levels but to female gender and serum C3 levels. The fact that this significant relationship between SI and female gender in our study was consistent with finding in a previous one^4^ suggests that sexual differentiation is related to SI irrespective of which organ is involved. The significant relationship noted between SI and serum C3 levels, which were not evaluated in the previous studies^4,5^, indicates that SI is unlikely to occur in patients with hypocomplementemia as a possible indicator of disease activity. Accordingly, a watchful observation without treatment may be appropriate to a certain extent in IgG4-RD patients with these characteristics in addition to asymptomatic and inactive disease.

On the other hand, observation without treatment may impose some degree of risk of irreversible organ dysfunction in IgG4-RD. Recent studies investigating its long-term clinical course have disclosed that dysfunction of affected organs can persist despite glucocorticoid treatment in pancreatic, renal, and salivary gland lesions^8–11^. In addition, such persistent organ dysfunction was reported to be related to existing organ dysfunction at the time of treatment initiation^10^ or a long interval between diagnosis and treatment initiation^11^, suggesting that delay of treatment initiation should be avoided. This makes it important to recognize the factors associated with deterioration during observation without treatment in addition to the factors related to SI mentioned above.

The present study showed that history of allergic disease, but not serum IgG4 elevation, was a factor significantly related to deterioration in treated IgG4-RD patients. This is consistent with the results of a recently published study by Liu et al.^7^ but inconsistent with those of the previous study investigating patients treated with rituximab^12^. Originally, an association between IgG4-RD and allergic diseases was suggested^1,13,14^. However, some more recent studies have indicated that allergic symptoms are not more frequent in IgG4-RD as compared to several control populations despite the common findings of serum IgE elevation and peripheral blood eosinophilia in the former^15–17^. Accordingly, any association of allergic predisposition with IgG4-RD pathogenesis or disease deterioration is yet unproven and remains to be clarified through further investigations.

On the other hand, interestingly, serum IgG4 elevation was the sole factor associated with deterioration in untreated patients in this study. A positive correlation between serum IgG4 levels and number of affected organs^18,19^ suggests that such untreated patients with higher serum IgG4 levels may have clinically silent affected organs and risk of future disease manifestations although whether an early treatment initiation for serum IgG4 elevation without obvious multiple organ manifestations is effective or not needs to be elucidated. More attention should be paid to deterioration of IgG4-RD in cases with high serum IgG4 levels disproportionate to the paucity of involved organs.

This study had several limitations. First, the treatment regimen and follow-up protocols were inconsistent among patients because of its retrospective nature, complicating evaluation of the influence of detailed treatment protocol differences on patient outcome. Second, although this study included more patients with salivary and ophthalmic lesions than past ones, the number of patients was not sufficient to conclude the identified factors to be definitively significant predictors. Therefore, larger-scale prospective studies will be needed to confirm our results.

In conclusion, the present study suggests that high serum IgG4 levels may be a useful predictor of the unfavorable outcomes of untreated patients with IgG4-RD, who tend to have fewer affected organs and lower IgG4-RD responder index. Although our results need to be confirmed through a larger-scale multicenter prospective study, the present observations may help to establish the optimal management strategy for IgG4-RD and in particular prevent treatment delay in untreated patients.

## Methods

### Patients and materials

We included 107 consecutive patients diagnosed with IgG4-RD between January 1, 2004, and December 31, 2017, who were followed-up for more than 6 months, at a single center in Japan. The diagnosis of IgG4-RD was made based on their fulfillment of the published comprehensive diagnostic criteria (CDC)^20^ or each set of organ-specific diagnostic criteria^21–23^ and exclusion of other diseases. Twenty-seven of these patients were followed up without treatment after the initial diagnosis. The following clinical factors at the time of diagnosis were retrospectively determined: age; gender; history of allergy; serum levels of IgG, IgG4, IgE, C3, C4, CH50, C-reactive protein (CRP), and creatinine; serum IgG4/IgE ratio^24^; peripheral blood eosinophil counts; presence of rheumatoid factor (RF) and anti-nuclear antibodies (ANA); number of affected organs; involvement of pancreas, salivary glands, ophthalmus, kidney, aorta/artery, retroperitoneum, and lung; IgG4-RD responder index. We compared the clinical features of these 27 patients with those of the 80 patients who underwent treatment. In addition, the patient outcomes were investigated, and logistic regression analysis was performed to assess factors related to the outcomes.

History of allergy was defined as past diagnosis of allergic diseases or conditions such as allergic rhinitis, bronchial asthma, urticaria, atopic dermatitis, and drug allergy, but not merely positive results of skin prick test or specific IgE measurement without clinical manifestations.

The IgG4-RD responder index is a disease activity assessment tool used to measure response to therapy in a structured manner^25,26^. It is conceptualized based on the Birmingham Vasculitis Activity Score for Wegener’s Granulomatosis. Using this index, investigators assess disease activity organ by organ and calculate the total score by summing all of the scores based on the individual organ assessments. Disease activity determined by the investigator reflects a patient’s symptoms attributable to active IgG4-RD and significant findings from the physical examination, laboratory examinations, and imaging studies.

Deterioration of IgG4-RD was defined as symptomatic, radiological, or functional exacerbation of the organ involved or de novo organ involvement. Spontaneous improvement (SI) of IgG4-RD was defined as symptomatic, radiological, or functional improvement of more than one of the organs involved and absence of deterioration as defined above in untreated patients.

This study received institutional ethics board approval from the Medical Ethics Committee of Kanazawa University, and informed consent for the use of all data and samples was obtained from each patient. We conducted the research in compliance with the Declaration of Helsinki.

### Statistical analysis

Statistical analysis was performed using SPSS V.25. Data are presented as median [interquartile range (IQR1, IQR3)] for continuous variables. The significance of differences between groups was determined using Mann–Whitney U test, while that of differences in frequencies was analyzed with Fisher’s exact probability test. For assessment of factors related to SI or deterioration of IgG4-RD during observation periods, unadjusted and age- and sex-adjusted logistic regression analyses were performed. Because SI or deterioration could frequently be detected only on imaging examinations, which were not always performed at frequent or regular intervals in this retrospective study, a time-to-event analysis such as Cox regression analysis was not used. In these logistic regression analyses, for continuous variables, unit for increments to calculate odds ratios were set at 1 year for age; 100 mg/dL for serum IgG4 and IgG levels; 100 IU/mL for serum IgE levels; 1 U/mL for serum CH50 levels; 1 mg/dL for serum C3, C4, CRP, and creatinine levels; 100 /µL for eosinophil counts. In addition, ROC curve analysis was performed to test the usefulness of certain parameters for the prediction of disease deterioration and to determine the appropriate cut-off value. Significant differences were defined as *P* < 0.05.

### Ethical approval and consent to participate

This study received institutional ethics board approval from the Medical Ethics Committee of Kanazawa University, and informed consent for the use of all data and samples was obtained from each patient. We conducted the research in compliance with the Declaration of Helsinki.

### Consent for publication

Not applicable.

## Data Availability

The data underlying this article will be shared on reasonable request to the corresponding author. The results presented in this paper have not been published previously in whole or part, except in abstract format.

## Abbreviations

AIP: Autoimmune pancreatitis
ANA: Anti-nuclear antibodies
CDC: Comprehensive diagnostic criteria
CI: Confidence interval
CRP: C-reactive protein
IgE: Immunoglobulin E
IgG: Immunoglobulin G
IgG4: Immunoglobulin G4
IQR: Interquartile range
IgG4-RD: Immunoglobulin G4 (IgG4)-related disease
OR: Odds ratio
RF: Rheumatoid factor
SI: Spontaneous improvement
SR: Spontaneous remissions
